# Left Ventricular Thrombi: Insights from Cardiac Magnetic Resonance Imaging

**DOI:** 10.3390/tomography7020016

**Published:** 2021-05-12

**Authors:** Narumol Chaosuwannakit, Pattarapong Makarawate

**Affiliations:** 1 Radiology Department, Faculty of Medicine, Khon Kaen University, Khon Kaen 40002, Thailand; 2 Cardiology Unit, Internal Medicine Department, Faculty of Medicine, Khon Kaen University, Khon Kaen 40002, Thailand; nchaosuw@gmail.com

**Keywords:** cardiac MRI, left ventricular thrombi, late gadolinium enhancement

## Abstract

*Objective*: Cardiovascular magnetic resonance imaging (CMR) late gadolinium enhancement technique (LGE) detects thrombus rather than anatomical presence based on tissue properties and is theoretically highly accurate. The present study’s goal was to compare the diagnostic accuracy obtained with various CMR techniques and transthoracic echocardiography to diagnose left ventricular thrombus and evaluate the prevalence and perspectives of left ventricular (LV) thrombus among patients with impaired systolic left ventricular function. *Methods*: In a single academic referral center, a retrospective database review of all CMR assessments of the established left ventricular thrombus was carried out in 206 consecutive patients with reduced systolic function for five years. To assess thrombus risk factors, clinical and imaging parameters were analyzed. Sensitivity, specificity, negative predictive value (NPV), positive predictive value (PPV), echocardiography, and cine-CMR sequence accuracy have been identified. LV structural parameters were quantified to detect markers for thrombus and predictors of the additive usefulness of contrast-enhanced thrombus imaging. Comparisons against LGE-CMR were made, which was used as the standard. *Results*: A 7.8 percent prevalence of left ventricular thrombus was identified by LGE-CMR. Cine-CMR increased the diagnostic efficiency for echocardiographic thrombus identification in this group, with sensitivity increasing from 50 percent by echocardiography to 75 percent by cine-CMR (*p* = 0.008). Dark blood CMR (DB-CMR) has better sensitivity and accuracy than echocardiography (*p* < 0.001), comparable to cine-CMR. The transmural infarct size was an independent marker for thrombus after correction for the LVEF and LV volume while considering only CMR parameters. There were significantly higher embolic events (HR = 71.33; CI 8.31–616.06, *p* < 0.0001) in LV thrombus patients detected by LGE-CMR. *Conclusion*: CMR imaging was more sensitive to left ventricular thrombi identification compared with transthoracic echocardiography. An additional parameter available from LGE-CMR and shown as an independent risk factor for left ventricular thrombus is the myocardial scar.

## 1. Introduction

There is considerable morbidity and mortality of left ventricular thrombus (LVT), complicating impaired left ventricular systolic function. LVT carries the possibility of embolic events in both the short and long terms [1,2]. In order to manage these patients, early diagnosis and initial management are crucial. Transthoracic echocardiography (TTE) in clinical practice is the first-line diagnostic tool for these patients. Nonetheless, previous studies have shown discordant findings found by echocardiography concerning thrombus prevalence identified by echocardiography.

Furthermore, discordant outcomes have been reported regarding the risk of future embolic events [3,4,5,6,7]. Prior echocardiography studies have identified substantial interobserver inconsistency in LVT diagnosis, with thrombus diagnosis being inconclusive in up to 46 percent of cases [8,9]. Moreover, echocardiography can be challenging for differentiating normal myocardium from LVT, hampering thin mural thrombus diagnosis. Although the standard transthoracic echocardiogram (TTE) is widely used for screening, its sensitivity for detecting LV thrombus is also limited. The detection of these thrombi with magnetic resonance imaging has a high yield. Anatomic (morphologic) evaluation using dark and bright blood sequences as well as cardiac function (motion) evaluation using bright-blood images of the beating heart are based on Steady-State Free Precession (SSFP). Myocardial viability (late gadolinium enhancement sequence) may provide additional information [10,11,12,13]. Currently, as a novel noninvasive cardiac imaging method, cardiac magnetic resonance imaging (CMR) has been introduced to complement data collected by echocardiography in patients with various cardiovascular diseases with plenty of other new CMR techniques to enhance sensitivity for LVT detection [10,11,12,13]. Late gadolinium enhancement cardiovascular magnetic resonance (LGE-CMR) has been reported to characterize viable and infarcted myocardium based on contrast uptake patterns. It has shown the ability to detect LV thrombus, a sensitive technique [11,12]. LGE-CMR can observe thrombus unnoticed by either cine-CMR or echocardiography [11,12]. The purpose of this study was to evaluate the prevalence of LGE-CMR thrombus in patients with an impaired left ventricular systolic function and to evaluate the diagnostic accuracy achieved with echocardiography and different CMR sequences for the diagnosis of LVT, as well as to evaluate different parameters for the prediction of the risk factor for LVT.

## 2. Materials and Methods

Between January 2012 and June 2016, the population consisted of impaired left ventricular systolic function patients who underwent transthoracic echocardiography and CMR at the Faculty of Medicine, Khon Kaen University, Thailand. Impaired systolic left ventricular was characterized as a quantitatively calculated left ventricular ejection fraction (LVEF) below 50 percent on cine-CMR. Two hundred and six consecutive patients undergoing TTE and CMR were enrolled in our research, administered by the local institutional oversight board under recommendations (HE601269). With state-of-the-art equipment, two-dimensional (2D) echocardiographic examinations were conducted using standard views and techniques following the American Society of Echocardiography guidelines. Images of patients in the left lateral position at end-expiration were acquired by experienced cardiologists.

**Figure 1:**
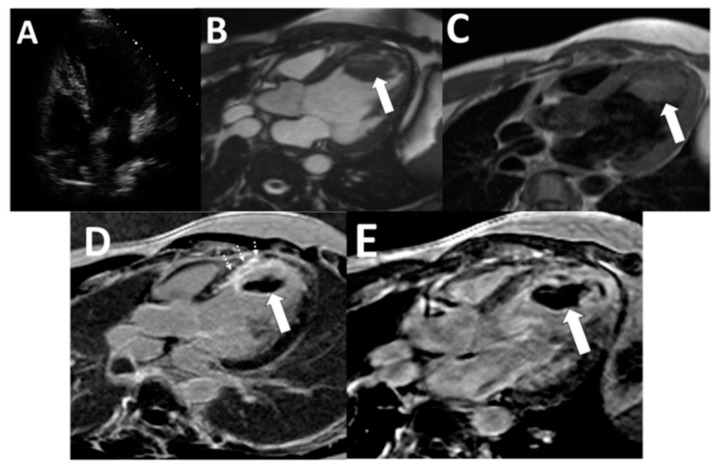
Examples of discordance between echocardiography and CMR. (**A**) Echocardiography was interpreted as negative. (**B**: arrow) LV apical thrombus demonstrated on cine-CMR, (**C**: arrow) DB-CMR, (**D**: arrow) standard LGE-CMR with an inversion time of 250–350 ms, and (**E**: arrow) long inversion time with inversion time = 600 ms. LGE-CMR. (**D**: dashed arrows) Myocardial scar at the anteroseptal segment of the left ventricle was demonstrated on LGE-CMR. Surgical resection enabled thrombus verification based on histopathology (not shown). (CMR: Cardiac magnetic resonance imaging, LGE-CMR: Late gadolinium enhancement sequence, DB-CMR: Dark blood sequence).

**Figure 2:**
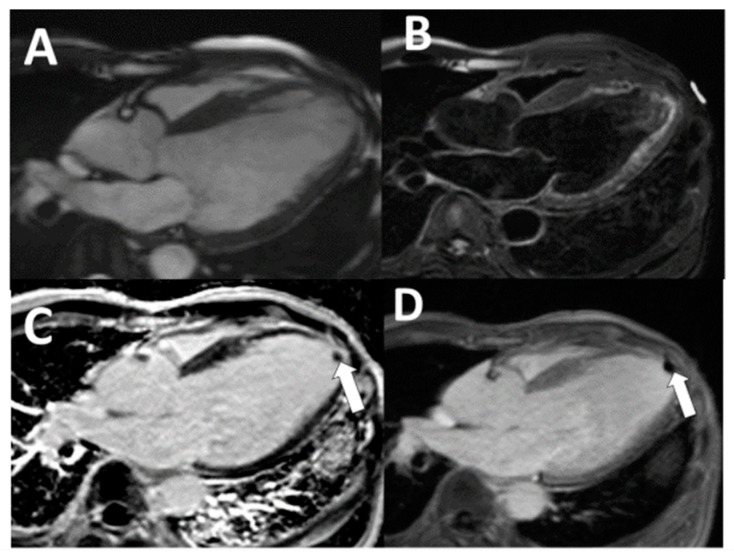
Example of false-negative cine-CMR and DB-CMR. Echocardiography was interpreted as negative (not shown). LV apical thrombus was not detected on (**A**) cine-CMR and (**B**) DB-CMR. LV apical thrombus was demonstrated on standard LGE-CMR with an inversion time of (**C**: arrow) 250–350 ms and (**D**: arrow) long inversion time with inversion time = 600 ms. This patient subsequently obtained antithrombotic medication after CMR. (CMR: Cardiac magnetic resonance imaging, LGE-CMR: Late gadolinium enhancement sequence, DB-CMR: Dark blood sequence).

For all the CMR imaging, a 1.5-T scanner (Siemens Medical Solutions, Erlangen, Germany) was used. In the CMR protocol, A single-shot turbo spin-echo (HASTE) sequence, dark blood CMR (DB-CMR), covering the whole heart in the axial direction, was used (flip angle 160°, TR/TE, two heartbeats/60 ms). A rapid imaging steady-state free precession (trueFISP) cine series (3 ms, 1.5 ms; flip angle 60° ms repetition time, 3.0 ms; echo time, 1.5 ms; in-plane spatial resolution, 1.7 × 1.4 mm; temporal resolution, 35 to 40 ms) was then acquired with standard cardiac views (cine-CMR). Images were collected to further examine questionable areas in the oblique orientation. Immediately after injection of 0.2 mmol/kg of gadolinium diethylenetriamine, four- and two-chamber heart views were acquired. In the short-axis direction, repetitive three-dimensional (3D) inversion recovery turbo FLASH sequences (4/1.4; flip angle 10°) were then executed. Images were acquired instantly after the contrast medium was injected and 15 min after injection (Late gadolinium enhancement CMR; LGE-CMR). While the 2D sequence is a single-slice technique (slice thickness, 8 mm), the 3D sequence of one breath-hold will obtain a slice thickness of 4 mm [13]. For LGE-CMR, inversion times were adjusted in the standard fashion to null viable myocardium [13]. In addition, a modified LGE-CMR sequence was designed for images with filling defects that were suspected of thrombus, in which the inversion time was increased from that required to null viable myocardium to a fixed time of 600 ms, which nulls avascular tissue such as thrombus [14]. Regions with a contrast uptake such as viable myocardium increase in image intensity with this long inversion time sequence (LGE-CMR long TI), thrombus appears homogeneously black, and thrombus delineation is improved (Figure 1 and Figure 2). Examples of discordance between echocardiography and CMR and example of false-negative cine-CMR and DB-CMR were demonstrated in Figure 1 and Figure 2. An experienced radiologist (level-3 qualified in CMR with more than ten years of experience interpreting CMR) interpreted all CMR images, unaware of the echocardiographic exam results. The DB-CMR, cine-CMR, and LGE-CMR, LGE-CMR long TI interpretations were conducted separately. LV thrombus was diagnosed using proven anatomical guidelines for cine-CMR and the established criteria for CMR [10,11,12,13]. CMR criteria for diagnosing LV thrombus were characterized as a non-enhancing mass within the LV cavity with distinct margins from the ventricular endocardium and distinguished from papillary muscles, chordae tendinae, trabeculations, or artifact. LV thrombus was evaluated on a thorough evaluation of both short- and long-axis views. On LGE-CMR, the LV thrombus was identified as a low-signal-intensity mass that could be distinguished from nearby high-intensity structures such as intracavity blood and myocardial scarring. The LV thrombus should appear in the LV cavity accompanied by features such as bulging structures and abrupt transitions [10,11,12,13].

### Statistical Analysis

Continuous variables were described as the mean ± standard deviation and categorical variables as the frequency (percentage). The sensitivity, specificity, negative predictive value (NPV), positive predictive value (PPV), and accuracy were reported. The diagnostic test performance and thrombus prevalence were compared using the McNemar test with exact binomial probability calculations. The magnitude of agreement between tests was measured using the kappa statistic (k). Multivariable logistic regression analyses were performed to evaluate the associations between imaging parameters and the thrombus. Statistical significance was considered representative of two-sided *p* < 0.05. The hazard ratio and Kaplan–Meier curve analysis for LV thrombus detected by LGE-CMR and embolic events were assessed. Analyses were conducted using version 19.0 of SPSS (SPSS Inc., Chicago, IL, USA).

## 3. Results

During the study period, 206 consecutive patients who underwent TTE and CMR were enrolled (age range, 23–76 years; mean age ± SD, 60.2 ± 14.2 years), and the baseline patient characteristics are shown in Table 1.

**Figure 3:**
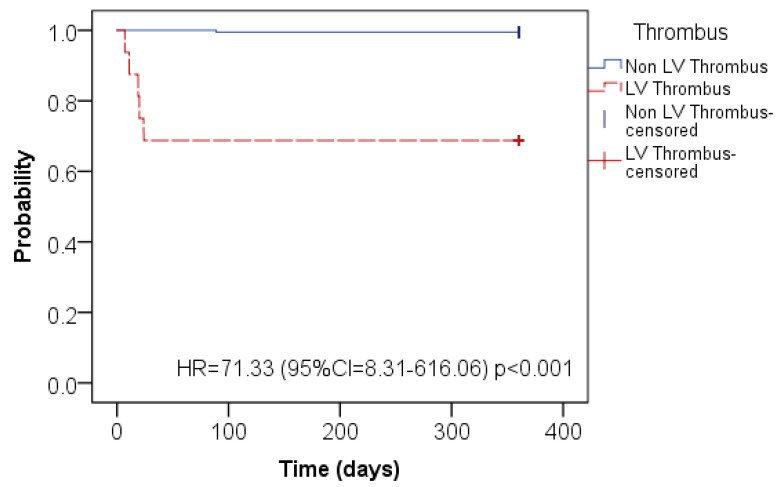
Kaplan–Meier curve for embolic events for the patients with LV thrombus detected by LGE-CMR (red line) and the patients who did not discover LV thrombus by LGE-CMR (blue line).

Advanced LV dysfunction (LVEF difference of 0.5 ± 6.9%, *p* = 0.42) was observed in both modalities. The majority of the individuals have a history of previous myocardial infarction (77.7%). At the time of CMR, 76% of patients were on antithrombotic medications; the most frequent indication was for suspected left ventricular thrombus or atrial fibrillation. Patients with LV thrombus detected by LGE-CMR had more embolic events than patients who did not discover LV thrombus by LGE-CMR (31.3% vs. 0.5%, HR = 71.33; CI 8.31–616.06, *p* < 0.0001) (Figure 3).

The median time for follow-up was six months (IQR 2–14 months). On the DB-CMR, five thrombi were not visible. Cine-CMR revealed evidence of advanced systolic dysfunction. By CMR, the mean LVEF was 39.9 ± 16.6%. In 8.7%, left ventricular aneurysms were present. LGE-CMR revealed 84 percent myocardial scarring; the mean scar size was 18.7 ± 11.6 percent of the overall LV myocardium. In 16 out of 206 patients, LGE-CMR showed LV thrombus (7.8%). Confirmation of left ventricular thrombus by pathology was obtainable in five patients, all of whom detected thrombus by LGE-CMR. As seen in Table 1, patients with thrombus did not differ significantly in age, history of previous myocardial infarction, or LV function from those without thrombus. Nonetheless, thrombus patients have a significantly higher incidence of LV aneurysm detected by echo (*p* < 0.001) and CMR (*p* < 0.001). In addition, as assessed by LGE-CMR, thrombus patients have had a larger infarct size (*p* < 0.001).

To determine the imaging parameters related to LV thrombus, multivariable tests were conducted to determine if LV infarction observed by LGE-CMR was an independent marker for thrombus after correction for cardiac function, chamber size, and morphology obtained either from echocardiography or from CMR. Transmural infarct size was an independent marker for thrombus after correction for the LVEF and LV volume while considering only CMR parameters (Table 2). The echocardiography diagnostic accuracy and different CMR techniques for diagnosing LV thrombus were compared with LGE-CMR as a reference. Cine-CMR sensitivity was 75 percent, and that of echocardiography in this population was just 50 percent (*p* = 0.008).

**Figure 4:**
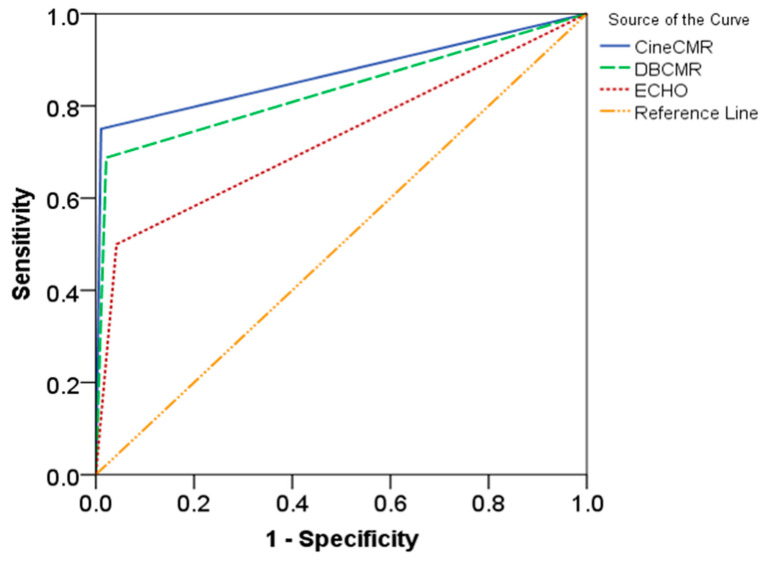
The receiver operating characteristic (ROC) curves of the left ventricular thrombi diagnostic performance from cine-CMR (solid blue line), DB-CMR (dashed green line), and echocardiography (dotted red line).

The areas under the ROC curve of echocardiography, cine CMR, and DB-CMR are 61.1% (95% confident interval of 57.1–88.7%), 87% (95% confident interval of 72.4–99.8%), and 72.9% (95% confident interval of 69.5–97.2%), respectively (Figure 4). Both Cine-CMR and DB-CMR had better sensitivity and accuracy (*p*< 0.001) than echocardiography. There were no significant differences between cine-CMR and DB-CMR in terms of diagnostic performance, with a good agreement between these modalities for thrombus diagnosis (Kappa = 0.78, *p* < 0.001). Echocardiography had a high specificity for the diagnosis of thrombus but low sensitivity and low positive predictive value (Table 3).

## 4. Discussion

The present study simultaneously compares echocardiography with other CMR techniques in order to detect LV thrombus, offering some new perspectives. First, 7.8 percent of patients showed the detection of LV thrombus by LGE-CMR in patients with a reduced LV systolic function. Second, LV thrombus identification by echocardiography had a low sensitivity, and cine-CMR and DB-CMR had similar diagnostic results with a higher sensitivity and accuracy compared to echocardiography (*p* < 0.001). Third, when comparing those who had LV thrombi detected on CMR to those who did not have thrombi detected on CMR, we observed a significant correlation between the prevalence of LV thrombi and embolic complications (31.3% vs. 0.5%, HR = 71.33; CI 8.31–616.06, *p* < 0.0001). Hence, LGE-CMR myocardial scarring was established as a novel risk factor for thrombus, and patients with LGE-CMR-diagnosed LV thrombus had more embolic occurrences than patients who did not detect LGE-CMR LV thrombus. Since LV thrombus is a substrate for thromboembolic events, LGE-CMR thrombus identification includes the possibility of improving clinical decision-making in patients with the impaired systolic function of LV. Of the 16 LV thrombus patients identified by LGE-CMR in the current study, 75% (12 of 16) identified LV thrombus using the cine-CMR technique.

A significant number out of certain patients, especially those with impaired LV function, continue to develop LV thrombi. The thrombus will provide an embolic substrate for subsequent embolic events. The initial diagnosis of these patients has generally been completed using echocardiography. Nevertheless, thrombi at the ventricular apex are usually unnoticed, and echocardiography may be limited or technically challenging as a mode of assessment.

As a result, an additional imaging modality, such as CMR imaging, is needed. In patients undergoing LV repair surgery, a previous study compared CMR with echocardiography [12]. This study demonstrates that transthoracic echocardiography sensitivity was at 23% compared to 88% for CMR [12]. Nevertheless, because LGE-CMR was analyzed in conjunction with cine-CMR, this study did not document any conclusions that should be drawn as to the importance of LGE-CMR for thrombus detection alone. Due to its wide availability, affordability, and high reliability, transthoracic echocardiography (TTE) is the first method of choice among the imaging techniques generally practiced to detect intracardiac thrombi. This technique is characterized by specificity values of about 90 percent for left ventricular thrombi but a low sensitivity compared to surgical or autopsy findings [15].

As a noninvasive method for detecting and comparing intracardiac mass from thrombus, cardiac MR imaging (CMR) with several sequences has recently emerged [16,17,18,19]. Due to the inadequate contrast between the thrombus and the myocardium and according to slow-flow artifacts, the five thrombi were not detectable on DB-CMR. This has impeded a correct diagnosis, especially in patients with impaired ventricular function or left ventricular aneurysms, comparable to previous studies [16,20]. Distinguishing a mural thrombus from the myocardium could be difficult because, on cine-CMR images, the thrombus exhibited a low signal intensity, so that the distinction between the myocardium and the thin mural thrombus was limited for cine-CMR [21]. Intravenous administration of LGE-CMR sequence gadolinium diethylenetriamine pentaacetic acid was found to improve the disparity between the myocardium and the thrombi, thus improving our ability to recognize and characterize thrombi [10,22]. On both traditional LGE-CMR and long inversion time LGE-CMR, the outline of thrombi was excellent. All thrombi appeared in the left ventricle as low-signal-intensity filling defects and remained at a dark signal intensity during the long LGE-CMR inversion time (Figure 1 and Figure 2). To validate CMR as a highly accurate technique for thrombus detection in competition with echocardiography, the present study was supported by the previous study using pathology and clinical evidence.

LGE-CMR is a commonly used technique to distinguish between viable and nonviable myocardium based on comparative differences in contrast uptake based on gadolinium and can be used for thrombus recognition [10,11,12,22]. While gadolinium-based contrast agents show uptake in infarcted myocardium, the thrombus is attributed to the absence of contrast uptake due to its avascular configuration [10]. Thrombus appears as a low-signal-intensity lesion on LGE-CMR in high-signal-intensity configurations, such as myocardium or intraventricular configurations. It is possible to use the absence of contrast enhancement to separate thrombus from other masses like neoplasm, which characteristically establishes contrast uptake. Thrombus appears gray, infarcted myocardium white, and viable myocardium black on conventional LGE-CMR tailored to null the viable myocardium. Both viable myocardium and thrombus may tend to be of relatively low signal intensity and can be difficult to distinguish against each other. By extending the inversion time to selectively null avascular thrombus, LGE-CMR can be supplementally optimized for thrombus assessment [10].

The current study had some limitations, despite the promising results, due to the retrospective study design, its single-center nature, and the comparatively limited sample size. Our findings could be verified by a more prospective analysis with a multicenter study.

## 5. Conclusions

With increased sensitivity and greater accuracy, cine-CMR improved the diagnostic efficiency for thrombus detection from echocardiography in this population. A comparable diagnostic performance was demonstrated by the left ventricular thrombus assessment using cine-CMR and DB-CMR. LGE-CMR-detected LV thrombus patients experienced substantially higher embolic events. In addition, a novel independent risk factor for LV thrombus was found to be a myocardial scar, as recognized by LGE-CMR.

## Figures and Tables

**Table 1:** Patient characteristics.

	All (*n* = 206)	Thrombus Present on LGE-CMR (*n* = 16)	Thrombus Absent on LGE-CMR (*n* = 190)	*p*-Value
**Age**	60.2 ± 14.2	62.5 ± 15.9	59.2 ± 13.1	0.34
**Male gender**	68.4% (141)	87.5% (14)	66.8% (127)	0.03
**Prior myocardial infarction**	77.7% (160)	87.5% (14)	76.8% (146)	0.65
**Coronary revascularization**	58.7% (121)	75% (12)	57.4% (109)	0.004
**History of embolic events**	2.9% (6)	31.3% (5)	0.5% (1)	<0.0001
**Antithrombotic medications such as aspirin, warfarin**	76.2% (157)	100% (16)	74.2% (141)	0.006
**Echocardiography**				
**LV function**				
**LVEF (%)**	40.5 ± 22.1	37.6 ± 18.9	41.1 ± 15.6	0.39
**End-diastolic diameter (cm)**	5.8 ± 0.8	5.9 ± 0.9	5.7 ± 1.0	0.44
**End-systolic diameter (cm)**	4.7 ± 0.9	4.9 ± 0.8	4.7 ± 1.0	0.34
**LV Aneurysm**	7.8% (16)	31.3% (5)	5.8% (11)	<0.001
**CMR**				
**LV function**				
**LVEF (%)**	39.9 ± 16.6	35.9 ± 11.5	41.5 ± 12.2	0.07
**End-diastolic volume (mL)**	189.2 ± 81.9	204.5 ± 99.8	169.9 ± 78.9	0.10
**End-systolic volume (mL)**	121.1 ± 99.1	137.6 ± 82.5	115 ± 79.6	0.28
**LV Aneurysm**	8.7% (18)	37.5% (6)	6.3% (12)	<0.001
**LV infarct size (% LV)**	18.7 ± 11.6	25.6 ± 9.8	14.6 ± 8.7	<0.001

**Table 2:** Multivariate Cox Regression analysis of LV thrombus.

Parameter	Regression Coefficient	Standard Error	*p*-Value
**LV end-diastolic volume**	205.6	99.9	0.42
**LV end-systolic volume**	139.8	77.8	0.58
**LV infarct size**	24.9	8.7	0.018

**Table 3:** Diagnostic performance of different techniques for detecting LV thrombus.

	Sensitivity (%) (95% CI)	Specificity (%) (95% CI)	Accuracy (%) (95% CI)	PPV (%) (95% CI)	NPV (%) (95% CI)
**Echocardiography**	50% (24.65–75.35)	96.84% (93.25–98.83)	93.09% (88.73–96.15)	57.93% (32.26–77.68)	95.7% (93.17–97.32)
**Cine-CMR**	75.0% (47.6–92.7)	98.95% (96.25–99.87)	97.03% (93.69–98.89)	86.1% (60.26–96.2)	97.85% (95.1–99.1)
**DB-CMR**	68.75% (41.3–88.9)	97.9% (94.7–99.4)	95.56% (91.78–97.94)	73.96% (50.48–88.78)	97.4% (94.57–97.94)

Note: Indexes were calculated using LGE-CMR as the standard for LV thrombus.

## Data Availability

Data sharing is not applicable to this article.
